# Macular Anatomy Differs in Dyslexic Subjects

**DOI:** 10.3390/jcm12062356

**Published:** 2023-03-17

**Authors:** Jose Javier Garcia-Medina, Nieves Bascuñana-Mas, Paloma Sobrado-Calvo, Celia Gomez-Molina, Elena Rubio-Velazquez, Maravillas De-Paco-Matallana, Vicente Zanon-Moreno, Maria Dolores Pinazo-Duran, Monica Del-Rio-Vellosillo

**Affiliations:** 1 Department of Ophthalmology, Optometry, Otolaryngology and Pathology, University of Murcia, 30100 Murcia, Spain; 2 General University Hospital Reina Sofia, 30003 Murcia, Spain; 3 General University Hospital Morales Meseguer, 30008 Murcia, Spain; 4 Ophthalmic Research Unit “Santiago Grisolia”, 46017 Valencia, Spain; 5 Spanish Net of Ophthalmic Pathology OFTARED RD16/0008/0022, Institute of Health Carlos III, 28029 Madrid, Spain; 6 Spanish Net of Inflammatory Diseases RICORS, Institute of Health Carlos III, 28029 Madrid, Spain; 7 Faculty of Health Sciences, International University of Valencia, 46002 Valencia, Spain; 8 Cellular and Molecular Ophthalmobiology Group, Surgery Department, Faculty of Medicine and Odontology, University of Valencia, 46010 Valencia, Spain; 9 University Hospital Virgen de la Arrixaca, 30120 Murcia, Spain; 10 Department of Surgery, Obstetrics and Gynecology and Pediatrics, University of Murcia, 30100 Murcia, Spain

**Keywords:** dyslexia, reading, retina, macula, fovea, parafovea, perifovea, optical coherence tomography, thickness, segmentation

## Abstract

The macula, as the central part of the retina, plays an important role in the reading process. However, its morphology has not been previously studied in the context of dyslexia. In this research, we compared the thickness of the fovea, parafovea and perifovea between dyslexic subjects and normal controls, in 11 retinal segmentations obtained by optical coherence tomography (OCT). With this aim, we considered the nine sectors of the Early Treatment Diabetic Retinopathy Study (ETDRS) grid and also summarized data from sectors into inner ring subfield (parafovea) and outer ring subfield (perifovea). The thickness in all the four parafoveal sectors was significantly thicker in the complete retina, inner retina and middle retina of both eyes in the dyslexic group, as well as other macular sectors (fovea and perifovea) in the inner nuclear layer (INL), inner plexiform layer (IPL), IPL + INL and outer plexiform layer + outer nuclear layer (OPL + ONL). Additionally, the inner ring subfield (parafovea), but not the outer ring subfield (perifovea), was thicker in the complete retina, inner retina, middle retina (INL + OPL + ONL), OPL + ONL, IPL + INL and INL in the dyslexic group for both eyes. In contrast, no differences were found between the groups in any of the sectors or subfields of the outer retina, retinal nerve fiber layer, ganglion cell layer or ganglion cell complex in any eye. Thus, we conclude from this exploratory research that the macular morphology differs between dyslexic and normal control subjects, as measured by OCT, especially in the parafovea at middle retinal segmentations.

## 1. Introduction

Dyslexia has been defined as a neurodevelopmental disorder characterized by reading difficulties in the absence of psychiatric, neurological, auditory or visual disabilities [1]. This disorder has been estimated to affect between 5 and 15% of children and around 4% of adults in the general population [2].

**Figure 1:**
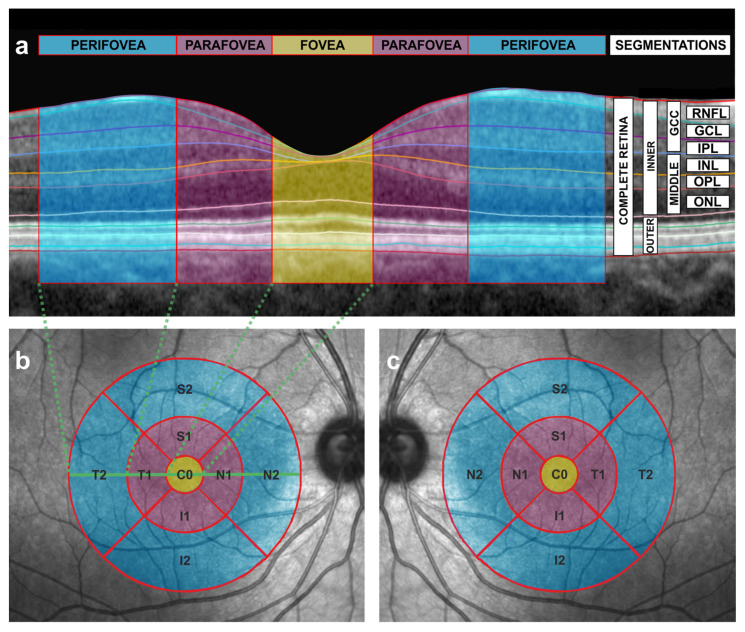
Cross-sectional and en face scans obtained by the means of optical coherence tomography (OCT). (**a**) Automatic segmentation of the different intraretinal layers in a cross-sectional OCT image of the macula from a right eye. The fovea is depicted in yellow, the parafoveal subfield in purple and the perifoveal subfield in blue. See Figure 1b,c for the corresponding en face representation of the fovea, parafovea and perifovea with the same color code as the one used in this figure. Dashed green lines indicate the limits of the temporal perifovea, the temporal parafovea and the fovea in relation to the en face image (see also Figure 1b). Segmentations are also shown. RNFL = retinal nerve fiber layer, GCL = ganglion cell layer (GCL), IPL= inner plexiform layer, INL = inner nuclear layer, OPL = outer plexiform layer, ONL = outer nuclear layer, GCC = ganglion cell complex, MIDDLE = middle retinal layers, INNER = inner retina, OUTER = outer retina. (**b**) Early Treatment Diabetic Retinopathy Study (ETDRS) grid with concentric circles of 1, 3 and 6 mm diameters of the right eye, showing nine sectors of the macula in an en face OCT image. The OCT device automatically estimates the mean thickness in microns for each sector and for each segmentation (see also Figure 1a). T = temporal, N = nasal, S = superior, I = inferior, C0 = fovea. Number 1 and number 2 refer to the inner ring and the outer ring, respectively, and correspond to the parafovea (inner ring) and the perifovea (outer ring). The macula is depicted with the same color code as in Figure 1a (the fovea in yellow, the parafovea in purple and the perifovea in blue). The horizontal, solid, green line indicates the location of the cross-sectional scan of Figure 1a in the en face image. Dashed green lines indicate the limits of the temporal perifovea, the temporal parafovea and the fovea in relation to cross-sectional images (see Figure 1a). (**c**) Early Treatment Diabetic Retinopathy Study (ETDRS) grid with concentric circles of 1, 3 and 6 mm diameters of the left eye, showing nine sectors of the macula in an en face OCT image. The OCT device automatically estimates the mean thickness in microns for each sector and for each segmentation (see also Figure 1a). T = temporal, N = nasal, S = superior, I = inferior, C0 = fovea. Number 1 and number 2 refer to the inner ring and the outer ring, respectively, and correspond to the parafovea (inner ring) and the perifovea (outer ring). The macula is depicted with the same color code as in Figure 1a (the fovea in yellow, the parafovea in purple and the perifovea in blue).

The pathophysiology of dyslexia is still controversial and has been attributed to phonological, auditory or visual alterations [3,4]. A number of neuroimaging investigations focused on the central nervous system (CNS) have been performed so far to study normal [5] and abnormal reading process [2,6], and also reading interventions [7,8]. However, we have to keep in mind that the first steps of a successful reading process are entirely visual and are related to the retina, a part of the CNS located in the eye [9]. The retina is made up of a complex cell circuitry of neurons (photoreceptors, bipolar cells, horizontal cells, amacrine cells and ganglion cells) and glial cells (astrocytes, Müller cells and microglial cells) that are arranged into alternating layers of the nuclei and axons/synapses [9,10]. These layers can be individually segmented in vivo in cross-sectional scans, and their thickness quantified with a non-invasive and reproducible technology called optical coherence tomography (OCT), that achieves a high resolution in cross-sectional images (Figure 1a), similar to that obtained in histological sections [11]. OCT technology has been extensively used to study biomarkers in other CNS disorders [12,13].

Specifically, the reading process starts with the projection of a well-focused image of the text onto the central part of the retina, called the macula. Then, this macular image is encoded by photoreceptors in a process called phototransduction, and transmitted through the chain of neurons of the visual pathway to the brain cortex to be interpreted [14]. The macular region includes the fovea, the parafovea and the perifovea [10] (Figure 1a–c).

Despite the macula being such an important site for the reading process, no study is available about the macular morphology in dyslexia as far as we know. Thus, the aim of this research was to compare the thickness of different retinal segmentations between dyslexic subjects and normal controls at the macula, by the means of OCT.

## 2. Materials and Methods

### 2.1. Recruitment 

In this study, dyslexic and normal controls were prospectively recruited from the patients attending the hospital for a routine ophthalmic review at the General University Reina Sofia Hospital of Murcia, Spain.

The inclusion criteria for the dyslexic group were: (1) Caucasian race; (2) Spanish as mother tongue; (3) aged under 25 years; (4) previous diagnosis of dyslexia confirmed by at least two different specialists; (5) refraction less than 6 spherical diopters and 2.5 cylinder diopters; (6) visual acuity of 20/25 or higher; (7) no history or findings of eye diseases or previous eye surgery; (8) no extraocular disease capable of modifying OCT measurements; (9) a reliable OCT scan (see below); (10) no other sensory, neurological, psychiatric, emotional or intellectual disorders; (11) no socio/economic significant disadvantage. Self-reported normal reader controls had the same inclusion criteria, except for criterion number 4. Only those participants not self-reporting reading difficulty and who correctly read aloud a simple 5-line paragraph text, without making a mistake or awkward pauses, were recruited in the control group.

### 2.2. Ophthalmic Examinations 

All the patients underwent a complete ophthalmic examination in both eyes, including visual acuity, autorefraction, air pneumatic tonometry, biomicroscopy and funduscopy. If the candidates were eligible, they underwent posterior pole horizontal protocol with 768 A- and 61 B-scans taken 30 × 25 degrees centered at the fovea, using a Spectralis OCT spectral domain device with an eye-tracking system (software version 6.0; Heidelberg Engineering, Heidelberg, Germany). 

The OCT examinations were performed in the morning, between 8:30 and 12:30 h, with pupil dilation. During this examination, the mean thickness of nine sectors was determined for each considered segmentation using the 1, 3, 6 mm diameter Early Treatment Diabetic Retinopathy Study protocol, that is one of the most used OCT en face patterns to study the macula, which considers the fovea, parafovea and perifovea subfields (Figure 1b,c). The position of the fovea was automatically determined by the device and checked by the same ophthalmologist (J.J.G.M.). Only reliable scans, with a signal strength over 20, were included. All the scans were performed by the same operator and inspected by the same experienced ophthalmologist (J.J.G.M.), in order to exclude eyes with segmentation errors, decentering or any other artifact. No manual corrections were made to the automatic segmentation performed by the prototype software. The examinations with decentrations, or with segmentation errors that could alter thickness estimation at any sector, were excluded. 

Then, with a segmentation tool (Segmentation Technology; Heidelberg Engineering), the thickness values of the following segmentations were automatically obtained: complete retina, inner retina, outer retina, retinal nerve fiber layer (RNFL), ganglion cell layer (GCL), inner plexiform layer (IPL), inner nuclear layer (INL), outer plexiform layer (OPL), outer nuclear layer (ONL). The thickness value of the other segmentations was also obtained by summing up the thicknesses of the automatically obtained segmentations as follows: ganglion cell complex (RNFL + GCL + IPL), middle retina (INL + OPL + ONL), IPL + INL and OPL + ONL (Figure 1a). The thicknesses of OPL and ONL were not considered individually and were summed up, as was performed in previous works regarding autism [15] and glaucoma [16,17,18], because these two layers are hard to be conveniently separated in OCT images due to their similar reflectivity. A three-dimensional video (Video S1) has been made in order to better understand the integration of cross-sectional (Figure 1a) and en face OCT images (Figure 1b,c). Considering that neurons and glia in the human retina are organized in concentric rings around the fovea [19,20], we also summarized the thickness for all the mentioned segmentations considering the inner ring subfield ((S1 + N1 + I1 + T1)/4) and the outer ring subfield ((S2 + N2 + I2 + T2)/4) that correspond to the parafovea (purple ring in Figure 1b,c) and to the perifovea (blue ring in Figure 1b,c), respectively.

### 2.3. Statistical Analysis

The data analysis was conducted using SPSS version 22.0 (SPSS, Inc., Chicago, IL, USA). As there were no previous similar studies, no sample size calculation was performed in this investigation. The results of the right and left eyes were separately compared between the groups. Gender was compared between the groups by a Fisher’s exact test. The quantitative variables were assessed for normality distribution by inspecting histograms and using the Shapiro–Wilk test. Normally distributed variables were expressed as the mean and standard deviation, while non-normally distributed values were expressed as the median and interquartile range. Comparisons between two normally distributed variables were performed with the unpaired Student’s t-test. If at least one variable was non-normally distributed, the comparison between the groups was made by the Mann–Whitney test. A correction for multiple comparisons was not applied to this study in order to avoid the false-negative results [21]. A *p*-value less than 0.05 was considered statistically significant.

## 3. Results

The OCT examinations of one right eye from the dyslexic group and two left eyes from the control group were discarded due to segmentation errors at one or more sectors. Finally, 89 reliable OCT scans from 46 participants were selected in this study: 24 right eyes (7 men, 17 women) and 25 left eyes (8 men, 17 women) were selected from 25 normal controls. Moreover, 21 right eyes (7 men, 14 women) and 19 left eyes (5 men, 14 women) were included from 21 dyslexic subjects. The gender between the dyslexic and control groups did not differ when considering the right (*p* = 1, Fisher’s exact test) or the left eyes (*p* = 0.749, Fisher’s exact test).

Similarly, the mean age was not different between the groups for the right eyes (15.83 ± 3.81 years for dyslexics, with a range of 9 to 23 years, and 16.00 ± 4.11 for normal controls, with a range of 10 to 23 years, *p* = 0.889, unpaired Student’s *t*-test) or for the left eyes (15.76 ± 3.75 years for dyslexics, with a range of 10 to 23 years, and 16.26 ± 3.97 for normal controls, with a range of 10 to 23 years, *p* = 0.672, unpaired Student’s *t*-test).

**Figure 2:**
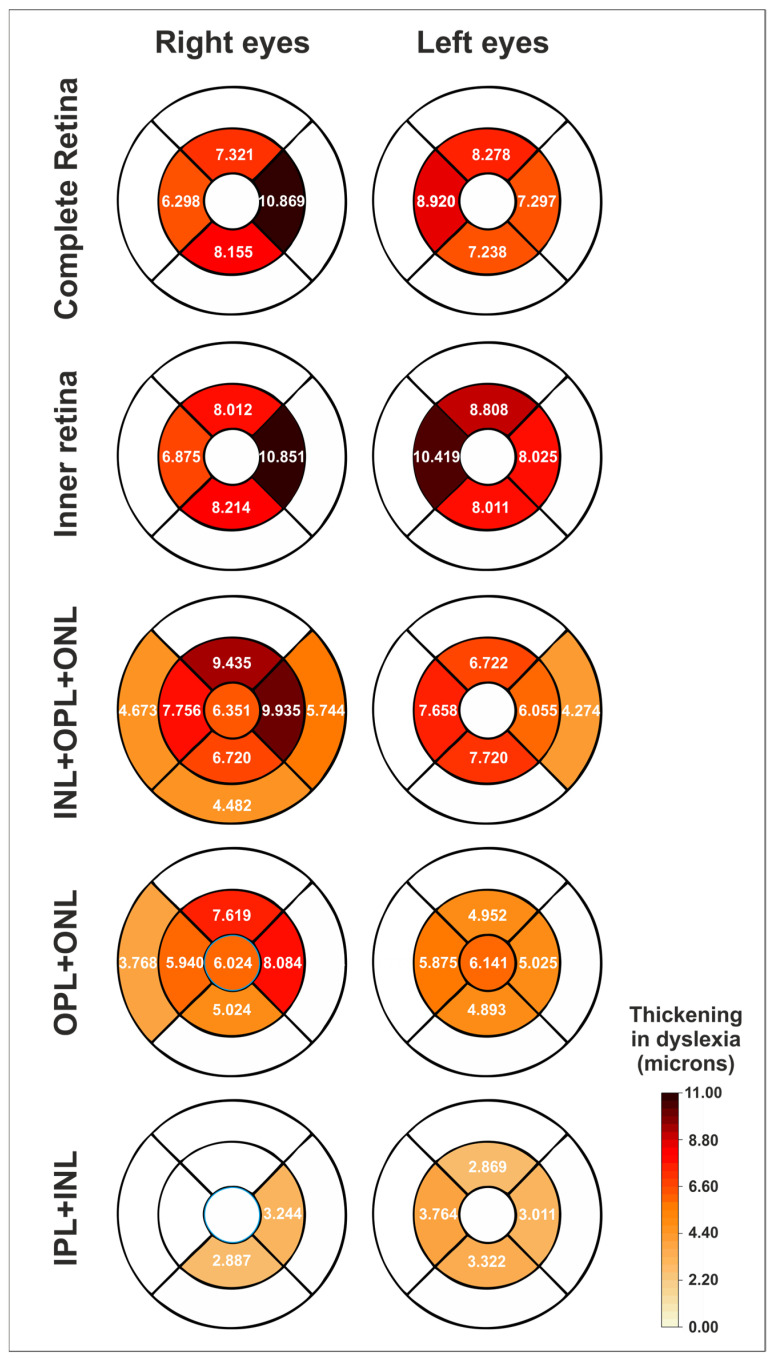
Schematic heatmaps of the statistically significant thickenings (expressed as microns) in the dyslexic group compared to the normal control group for the right and left eyes (mean differences in thickness) and for different segmentations. See also Figure 1a–c. White sectors indicate no significant differences. IPL = inner plexiform layer, INL = inner nuclear layer, OPL = outer plexiform layer, ONL = outer nuclear layer. See also Tables S1–S4 and S7.

The thicknesses in all four parafoveal sectors were significantly thicker in both the right and left eyes of the dyslexic group in the following segmentations: complete retina (Table S1), inner retina (Table S2), middle retina (INL + OPL + ONL) (Table S3) and OPL + ONL (Table S4). Several macular sectors were also thicker in IPL (Table S5), INL (Table S6) and IPL + INL (Table S7) in dyslexia. Moreover, a foveal thickening was also observed in both eyes for OPL + ONL (and also for INL + ONL + OPL in the right eye) (Figure 2).

In contrast, no thickness differences were observed between both the groups in any of the sectors of the retinal nerve fiber layer (RNFL) (Table S8), ganglion cell layer (GCL) (Table S9), ganglion cell complex (RNFL + GCL + IPL) (Table S10) or outer retina (Table S11) in either eye. 

When considering the inner ring subfield (parafovea), we found that the complete retina, inner retina, middle retina (INL + OPL + ONL), OPL + ONL, IPL + INL and INL showed a higher thickness in the dyslexic group for both eyes, with IPL showing this difference only for the right eye (Table 1). The rest of the segmentations (outer retina, GCC, RNFL, GCL) did not present any significant differences in either eye (Table 1).

In contrast, when dealing with the outer ring subfield (perifovea), only INL + OPL + ONL and INL showed a discrete increase of thickness in the dyslexia group, only when comparing the right eyes (Table 2).

## 4. Discussion

This study found parafoveal thickenings in both eyes of the dyslexic group for the middle retina, mainly the ONL-related segmentations, but also for the INL-related segmentations (Table 1). Remarkably, no difference was noted in the GCL-related segmentations or in the outer retina of right or left eyes. Furthermore, complete retina and inner retinal thickenings found at the parafovea in this study seem to be due to the middle retinal thickenings (Figure 1a; see also Table 1 and Tables S1–S4 and S7). Additionally, a foveal thickening was also observed in ONL + OPL (in both eyes) and INL + ONL + OPL (in the right eye).

The ONL contains photoreceptor cell bodies and the INL bipolar, amacrine and horizontal cells nuclei. OPL and IPL are made up of axons and synapses and connect the neighboring nuclear layers (ONL-INL and INL-GCL, respectively) [9]. In fact, the ONL and INL layers share a common embryological origin: the outer neuroblastic zone differentiates into ONL and INL after fetal week 10. Then, first synapses appear in the IPL and OPL by fetal week 12 [10]. ONL and INL the somata are displaced during normal foveal development (from fetal week 22 to postpartum month 45): photoreceptor cell bodies (ONL) present a centripetal displacement toward the foveal center with cellular packing and elongation, while INL and GCL have centrifugal displacement to the foveal rim [10]. One possible explanation for the thickenings found in this study is a disorder in foveal development due to a gap between these two movements in the opposite direction. In this way, orientation of the somata and the axons of ONL- and INL-related cells could result in more vertical increasing of the thickness of these layers. This mechanism has been proposed to explain some similar retinal features found in the eyes of preterm patients with foveal immaturity [22]. Other alternative explanations for the found thickenings could be a neuronal/glial population increase, cell size augmentation or an extracellular expansion in the affected segmentations. More studies are warranted to elucidate this question. 

A genetically determined disorder in foveal development could bring about the findings of the present study. This hypothesis is consistent with the fact that dyslexia presents a high degree of heritability (70% or even more), whereas the environment has little effect [23,24,25].

As a matter of fact, some genetic polymorphisms have been detected to be much more prevalent in dyslexia. These polymorphisms are in relation to abnormal axonal growth and defective neural migration [26,27,28], and have been associated with alterations in the development of cortico-cortical and cortico-thalamic circuits in dyslexia [2,29], so they may similarly lead to the retinal differences detected in this study. 

The highest thickenings found in this study were located at the parafovea, although alterations at the fovea, and even at some perifoveal sectors, were also present in OPL + ONL and INL + OPL + ONL segmentations (Figure 2). The fovea captures the visual field one degree around the fixation point, while the parafovea is surrounding the fovea up to five degrees from the fixation point [30,31]. 

The fovea and the parafovea work together in the reading process, because, although the fovea is oriented to the target word, the parafovea previews the next words to facilitate further foveal processing [32,33]. In fact, parafoveal recognition of embedded letters and words has been proven to be worse in dyslexic subjects than in normal controls [34,35,36]. Furthermore, reduced and delayed parafoveal preview benefits have been associated with dyslexia [37,38,39,40]. 

Thus, the thickenings found in this study could constitute the morphological correlation of parafoveal dysfunction in dyslexia. These thickenings may theoretically cause a higher level of light scattering transmitted to the outer segments of photoreceptors, where the phototransduction takes place, and potentially a subsequent loss of sensitivity [10]. The other possibility is that the found thickenings could be related to a change in the arrangement of the photoreceptors and/or Müller cells, as suggested above. An incorrect arrangement of these cells may induce an incorrect angle of incidence of light and a subsequent reduction of sensitivity due to the Stiles–Crawford effect of the first kind [41,42]. These, or other causes associated to these thickenings, may alter parafoveal preview function. Further study is required in this sense.

It is also remarkable that the thickening in all four parafoveal sectors (360 degrees) found herein is consistent with the fact that dyslexia is present in all languages, independently of the reading direction going from left to right (English, Spanish, French, German), from right to left (Hebrew or Arabic) or from top to bottom (Japanese, Chinese) [6].

This exploratory research also has its limitations. First, the sample size is small and the outcomes should be studied in larger groups, but we found significant results. Moreover, the facts that the comparisons have been calculated separately for the right and the left eyes and that the found differences are so similar in both eyes, affecting the same segmentations and with analogous disparities, reinforce the validity of our results (Figure 2). Second, the participants included in this study are mainly adolescents and young adults, so we cannot extrapolate our results to other age groups. Third, this study is cross-sectional, so we cannot know whether the found differences are stable over time and whether reading interventions are able to remodel retinal structures, similarly to what has been found in other investigations on the CNS in dyslexia [7,8]. Fourth, the grouping of this study is based on a diagnosis and not on specific reading measurements. Using a categorical method to create the groups does not permit to explore a quantitative correlation between reading measurements (for example, reading speed) and OCT parameters. However, we have to keep in mind that this is an exploratory investigation. Further works should deal with these relationships. Fifth, it should be pointed out that the nomenclature of the inner and outer retina, as defined by the OCT device used in this study (Spectralis, Heidelberg Engineering), is inexact (Figure 1). The inner and outer retina are major divisions in both neurobiology and vascular biology. The outer retina includes the OPL, ONL, photoreceptors and retinal pigment epithelium, and receives blood supply from choroidal circulation, whereas the inner retinal layers are dependent on retinal circulation [43]. However, this fact does not affect the results of this study. Sixth, this study is limited in analyzing morphological results. Further functional studies (i.e., mERG) in relation to morphological differences should be investigated in further studies. Seventh, the OCT examinations were only analyzed by one expert, so inter-variability agreement cannot be assessed. However, all the examinations fulfilled the reliability criteria. Finally, this study is limited to describe morphological differences in the macula of dyslexic subjects, so we cannot determine if these findings are causes or consequences, or the parallel manifestations in the pathophysiology of dyslexia: if these morphological findings were the cause, dyslexia could be primarily a retinal disorder, as suggested above. If they were the consequence or a parallel manifestation, dyslexia would be capable of remodeling retinal structures, as is done in other CNS structures [2,6,44]. Thus, the macula could be a privileged and accessible site to study dyslexia by using a fast, inexpensive and non-invasive technique, such as OCT. Further studies are required in this sense. Nevertheless, our significant results open a new horizon for the investigation of dyslexia.

## 5. Conclusions

From this exploratory research, we conclude that the macular morphology differs in dyslexic and normal controls, especially in the parafovea. 

## Figures and Tables

**Table 1:** Thickness comparison between the groups for the inner ring subfield (parafovea) in the ETDRS grid.

Thickness of the Inner ETDRS Ring Subfield (Parafovea) Comparisons between Normal Controls and Dyslexic Subjects									
		Right Eyes (24 vs. 21)				Left Eyes (25 vs. 19)			
	Group	Mean	Std. Dev.	Mean Dif.	*p* (UTT)	Mean	Std. Dev.	Mean Dif.	*p* (UTT)
**Complete retina**	Control	330.63	10.25	−8.16	**0.011**	330.33	10.36	−7.93	**0.019**
	Dyslexia	338.79	10.32			338.26	11.04		
**Inner retina**	Control	251.08	8.72	−8.49	**0.004**	250.50	9.11	−8.82	**0.006**
	Dyslexia	259.57	10.07			259.32	10.98		
**Outer retina**	Control	79.48	2.12	0.30	0.599	79.87	2.46	0.94	0.176
	Dyslexia	79.18	1.60			78.93	1.89		
**GCC**	Control	114.38	5.52	0.58	0.777	113.29	4.60	−1.89	0.278
	Dyslexia	113.80	7.97			115.18	6.82		
**INL + OPL + ONL**	Control	137.31	6.73	−8.46	**0.0002**	137.38	6.51	−6.87	**0.003**
	Dyslexia	145.77	7.67			144.25	7.70		
**ONNL**	Control	98.19	5.73	−6.66	**0.001**	98.45	5.63	−5.18	**0.010**
	Dyslexia	104.85	6.94			103.63	7.07		
**IPL + INL**	Control	80.28	3.70	−2.55	**0.036**	80.14	3.86	−3.24	**0.009**
	Dyslexia	82.83	4.22			83.38	3.92		
**RNFL**	Control	21.01	2.59	0.99	0.142	20.24	1.33	0.17	0.681
	Dyslexia	20.02	1.66			20.07	1.45		
**GCL**	Control	52.21	2.71	0.34	0.758	51.84	2.67	−0.52	0.595
	Dyslexia	51.87	4.33			52.36	3.71		
**IPL**	Control	41.16	1.67	−0.74	0.310	41.21	1.76	−1.55	**0.015**
	Dyslexia	41.90	2.93			42.76	2.31		
**INL**	Control	39.13	2.61	−1.80	**0.020**	38.93	2.62	−1.69	**0.025**
	Dyslexia	40.93	2.34			40.62	2.05		

Right and left eyes were independently compared between the groups. The thickness results are expressed as microns. Statistically significant results are depicted in bold. Std. Dev = standard deviation, Dif. = difference, UTT = unpaired *t*-test, GCC = ganglion cell complex, RNFL = retinal nerve fiber layer, GCL = ganglion cell layer, IPL = inner plexiform layer, INL = inner nuclear layer, OPL = outer plexiform layer, ONL = outer nuclear layer.

**Table 2:** Thickness comparison between the groups for the outer ring subfield (perifovea) in the ETDRS grid.

Thickness of the Outer ETDRS Ring Subfield (Perifovea) Comparisons between Normal Controls and Dyslexic Subjects									
		Right Eyes (24 vs. 21)				Left Eyes (25 vs. 19)			
	Group	Mean	Std. Dev.	Mean Dif.	*p* (UTT)	Mean	Std. Dev.	Mean Dif.	*p* (UTT)
**Complete retina**	Control	294.82	12.43	−4.66	0.256	294.38	10.64	−4.57	0.218
	Dyslexia	299.48	14.67			298.95	13.62		
**Inner retina**	Control	217.85	11.48	−4.35	0.271	217.14	9.97	−4.91	0.173
	Dyslexia	222.20	14.62			222.05	13.54		
**Outer retina**	Control	76.91	1.73	−0.42	0.380	77.20	1.67	0.29	0.564
	Dyslexia	77.33	1.46			76.91	1.62		
**GCC**	Control	100.31	6.96	−0.58	0.808	100.31	6.96	−0.58	0.804
	Dyslexia	100.89	8.60			100.89	8.60		
**INL + OPL + ONL**	Control	117.07	5.84	−4.58	**0.020**	117.30	5.81	−3.81	0.057
	Dyslexia	121.65	6.90			121.11	7.06		
**ONP + ONL**	Control	83.07	5.03	−3.31	0.051	83.27	4.97	−2.93	0.083
	Dyslexia	86.38	6.02			86.20	5.96		
**IPL + INL**	Control	63.64	3.42	−1.82	0.132	63.66	3.60	−1.71	0.165
	Dyslexia	65.46	4.55			65.37	4.41		
**RNFL**	Control	34.81	5.25	1.30	0.331	33.79	3.99	0.41	0.718
	Dyslexia	33.51	3.23			33.38	3.24		
**GCL**	Control	36.98	2.95	0.06	0.956	36.89	2.75	−0.16	0.871
	Dyslexia	36.92	4.38			37.05	3.87		
**IPL**	Control	29.64	1.97	−0.55	0.471	29.63	1.71	−0.83	0.258
	Dyslexia	30.19	2.97			30.46	2.75		
**INL**	Control	34.00	1.76	−1.27	**0.020**	34.03	2.23	−0.88	0.171
	Dyslexia	35.27	1.78			34.91	1.84		

Right and left eyes were independently compared between the groups. The thickness results are expressed as microns. Statistically significant results are depicted in bold. Std. Dev. = standard deviation, Dif. = difference, UTT = unpaired *t*-test, GCC = ganglion cell complex, RNFL = retinal nerve fiber layer, GCL = ganglion cell layer, IPL = inner plexiform layer, INL = inner nuclear layer, OPL = outer plexiform layer, ONL = outer nuclear layer.

## Data Availability

The data sets generated and/or analyzed during this study are available from the corresponding author on reasonable request.

## Supplementary Materials

The following supporting information can be downloaded at: https://www.mdpi.com/article/10.3390/jcm12062356/s1, Video S1 and Tables S1–S11 have been added as supplementary material. Video S1. Three-dimensional (3D) video that dynamically combines the cross-sectional and the en face OCT scans of the macula from a right eye. The cross-sectional scans that configure the 3D surface of the macula progressively disappear, while the two-dimensional image of the en face scan remains. See also Figure 1a,b.
