# Supplementary material for: Macular Anatomy Differs in Dyslexic Subjects
Source: J Clin Med. 2023 Mar 17;12(6):2356. doi: 10.3390/jcm12062356 (PMC10057708; doi:10.3390/jcm12062356)
Supplement: Supplementary file 1 [file jcm-12-02356-s001.zip › supplementary tables S1-S11.pdf]

Complete retina. Comparisons between groups (Right eyes)								
	**Group**	**n**	**Mean**	**Std. dev.**	**Dif. Mean**	**Median**	**IQR**	**Significance (test)**
**C0**	Control	24	N/A	N/A	N/A	254.50	17	.417 (MWT)
	Dyslexia	21	263.67	18.076		260.00	18	
**N1**	Control	24	333.75	11.939	-10.869	N/A	N/A	**.003** (UTT)
	Dyslexia	21	344.62	11.373		N/A	N/A	
**N2**	Control	24	314.71	14.085	-5.482	N/A	N/A	.238 (UTT)
	Dyslexia	21	320.19	16.669		N/A	N/A	
**S1**	Control	24	334.92	11.424	-7.321	N/A	N/A	**.032** (UTT)
	Dyslexia	21	342.24	10.573		N/A	N/A	
**S2**	Control	24	295.58	12.169	-5.607	N/A	N/A	.176 (UTT)
	Dyslexia	21	301.19	15.155		N/A	N/A	
**T1**	Control	24	321.75	9.830	-6.298	N/A	N/A	**.029** (UTT)
	Dyslexia	21	328.05	8.657		N/A	N/A	
**T2**	Control	24	279.71	11.331	-4.720	N/A	N/A	.190 (UTT)
	Dyslexia	21	284.43	12.440		N/A	N/A	
**I1**	Control	24	332.08	10.202	-8.155	N/A	N/A	**.019** (UTT)
	Dyslexia	21	340.24	12.165		N/A	N/A	
**I2**	Control	24	289.29	15.892	-2.804	N/A	N/A	.572 (UTT)
	Dyslexia	21	292.10	17.097		N/A	N/A	
**Complete retina. Comparisons between groups (Left eyes)**								
	**Group**	**n**	**Mean**	**Std. dev.**	**Dif. Means**	**Median**	**IQR**	**Significance (test)**
**C0**	Control	25	N/A	N/A	N/A	255.00	21	.740 (MWT)
	Dyslexia	19	262.00	18.166		258.00	18	
**N1**	Control	25	335.08	11.150	-8.920	N/A	N/A	**.014** (UTT)
	Dyslexia	19	344.00	11.902		N/A	N/A	
**N2**	Control	25	314.56	10.595	-5.598	N/A	N/A	.212 (UTT)
	Dyslexia	19	320.16	16.724		N/A	N/A	
**S1**	Control	25	334.88	11.337	-8.278	N/A	N/A	**.028** (UTT)
	Dyslexia	19	343.16	12.742		N/A	N/A	
**S2**	Control	25	297.72	12.225	-4.648	N/A	N/A	.254 (UTT)
	Dyslexia	19	302.37	14.438		N/A	N/A	
**T1**	Control	25	320.44	10.288	-7.297	N/A	N/A	**.021** (UTT)
	Dyslexia	19	327.74	9.527		N/A	N/A	
**T2**	Control	25	278.68	11.298	-5.846	N/A	N/A	.109 (UTT)
	Dyslexia	19	284.53	12.281		N/A	N/A	
**I1**	Control	25	330.92	10.688	-7.238	N/A	N/A	**.039** (UTT)
	Dyslexia	19	338.16	11.730		N/A	N/A	
**I2**	Control	25	286.56	11.288	-2.177	N/A	N/A	.563 (UTT)
	Dyslexia	19	288.74	13.490		N/A	N/A	

Table S1. Thickness comparison between groups for the complete retina in the ETDRS grid. Right and left eyes were separately compared. T=Temporal, N=nasal, S=superior, I=Inferior, C0=fovea. Number 1 and number 2 refer to the inner ring and the outer ring, respectively, and correspond to the parafovea (inner ring) and the perifovea (outer ring). n=number, Std. Dev=standard deviation, Dif.=Difference, IQR=interquartile range, UTT= Unpaired t-test MWT=Mann-Whitney test, N/A=not applicable. The thickness results are expressed as microns. Statistically significant results are depicted in bold.

Inner retina. Comparisons between groups (Right eyes)								
	**Group**	**n**	**Mean**	**Std. dev.**	**Mean Dif.**	**Median**	**IQR**	**Significance (test)**
**C0**	Control	24	N/A	N/A	N/A	165.5	19	.168 (MWT)
	Dyslexia	21	178.00	18.754		175.0	19	
**N1**	Control	24	252.96	10.675	-10.851	N/A	N/A	**.002** (UTT)
	Dyslexia	21	263.81	11.285		N/A	N/A	
**N2**	Control	24	236.58	13.065	-5.274	N/A	N/A	.240 (UTT)
	Dyslexia	21	241.86	16.590		N/A	N/A	
**S1**	Control	24	255.42	9.824	-8.012	N/A	N/A	**.011** (UTT)
	Dyslexia	21	263.43	10.443		N/A	N/A	
**S2**	Control	24	218.25	11.558	-5.083	N/A	N/A	.221 (UTT)
	Dyslexia	21	223.33	15.809		N/A	N/A	
**T1**	Control	24	242.46	8.241	-6.875	N/A	N/A	**.008** (UTT)
	Dyslexia	21	249.33	8.345		N/A	N/A	
**T2**	Control	24	203.29	10.348	-4.708	N/A	N/A	.172 (UTT)
	Dyslexia	21	208.00	12.377		N/A	N/A	
**I1**	Control	24	253.50	8.753	-8.214	N/A	N/A	**.011** (UTT)
	Dyslexia	21	261.71	11.921		N/A	N/A	
**I2**	Control	24	213.29	14.959	-2.327	N/A	N/A	.623 (UTT)
	Dyslexia	21	215.62	16.527		N/A	N/A	
**Inner retinal. Comparisons between groups (Left eyes)**								
	**Group**	**n**	**Mean**	**Std. dev.**	**Mean Dif.**	**Median**	**IQR**	**Significance (test)**
**C0**	Control	25	N/A	N/A	N/A	166.00	22	.387 (MWT)
	Dyslexia	19	176.05	17.995		173.00	21	
**N1**	Control	25	253.16	9.919	-10.419	N/A	N/A	**.003** (UTT)
	Dyslexia	19	263.58	12.107		N/A	N/A	
**N2**	Control	25	236.12	9.799	-6.354	N/A	N/A	.150 (UTT)
	Dyslexia	19	242.47	16.645		N/A	N/A	
**S1**	Control	25	255.56	10.251	-8.808	N/A	N/A	**.015** (UTT)
	Dyslexia	19	264.37	12.707		N/A	N/A	
**S2**	Control	25	219.96	11.667	-4.829	N/A	N/A	.240 (UTT)
	Dyslexia	19	224.79	15.241		N/A	N/A	
**T1**	Control	25	241.08	8.841	-8.025	N/A	N/A	**.006** (UTT)
	Dyslexia	19	249.11	9.279		N/A	N/A	
**T2**	Control	25	202.16	10.168	N/A	201.00	18	.790 (MWT)
	Dyslexia	19	N/A	N/A		207.00	23	
**I1**	Control	25	252.20	9.570	-8.011	N/A	N/A	**.016** (UTT)
	Dyslexia	19	260.21	11.679		N/A	N/A	
**I2**	Control	25	210.32	10.984	-2.259	N/A	N/A	.534 (UTT)
	Dyslexia	19	212.58	12.907		N/A	N/A	

Table S2. Thickness comparison between groups for the inner retina in the ETDRS grid. Right and left eyes were independently compared. T=temporal, N=nasal, S=superior, I=inferior, C0=fovea. Number 1 and number 2 refer to the inner ring and the outer ring, respectively, and correspond to the parafovea (inner ring) and the perifovea (outer ring). n=number, Std. Dev=standard deviation, Dif.=Difference, IQR=interquartile range, UTT= Unpaired t-test MWT=Mann-Whitney test, N/A=not applicable. The thickness results are expressed as microns. Statistically significant results are depicted in bold.

Middle retinal layers (INL+OPL+ONL). Comparisons between groups (Right eyes)								
	**Group**	**n**	**Mean**	**Std. dev.**	**Mean Dif.**	**Median**	**IQR**	**Significance (test)**
**C0**	Control	24	126.79	7.785	-6.351	N/A	N/A	**.030**
	Dyslexia	21	133.14	11.096		N/A	N/A	(UTT)
**N1**	Control	24	139.88	7.116	-9.935	N/A	N/A	**.0001**
	Dyslexia	21	149.81	8.892		N/A	N/A	(UTT)
**N2**	Control	24	120.54	6.507	-5.744	N/A	N/A	**.011**
	Dyslexia	21	126.29	8.076		N/A	N/A	(UTT)
**S1**	Control	24	138.04	6.702	-9.435	N/A	N/A	**.001**
	Dyslexia	21	147.48	10.769		N/A	N/A	(UTT)
**S2**	Control	24	119.67	5.858	-3.429	N/A	N/A	.106
	Dyslexia	21	123.10	8.037		N/A	N/A	(UTT)
**T1**	Control	24	135.96	6.906	-7.756	N/A	N/A	**.001**
	Dyslexia	21	143.71	6.922		N/A	N/A	(UTT)
**T2**	Control	24	117.04	6.464	-4.673	N/A	N/A	**.020**
	Dyslexia	21	121.71	6.497		N/A	N/A	(UTT)
**I1**	Control	24	135.38	7.149	-6.720	N/A	N/A	**.004**
	Dyslexia	21	142.10	7.516		N/A	N/A	(UTT)
**I2**	Control	24	111.04	6.868	-4.482	N/A	N/A	**.042**
	Dyslexia	21	115.52	7.467		N/A	N/A	(UTT)
**Middle retinal layers (INL+OPL+ONL). Comparisons between groups (Left eyes)**								
	**Group**	**n**	**Mean**	**Std. dev.**	**Mean Dif.**	**Median**	**IQR**	**Significance (test)**
**C0**	Control	25	N/A	N/A	N/A	123.00	10	.061
	Dyslexia	19	131.63	11.142		132.00	14	(MWT)
**N1**	Control	25	139.92	6.519	-7.659	N/A	N/A	**.002**
	Dyslexia	19	147.58	8.637		N/A	N/A	(UTT)
**N2**	Control	25	120.60	5.723	-4.032	N/A	N/A	.073
	Dyslexia	19	124.63	8.802		N/A	N/A	(UTT)
**S1**	Control	25	139.12	7.418	-6.722	N/A	N/A	**.008**
	Dyslexia	19	145.84	8.636		N/A	N/A	(UTT)
**S2**	Control	25	120.44	6.430	-3.876	N/A	N/A	.083
	Dyslexia	19	124.32	8.070		N/A	N/A	(UTT)
**T1**	Control	25	135.84	6.908	-6.055	N/A	N/A	**.007**
	Dyslexia	19	141.89	7.172		N/A	N/A	(UTT)
**T2**	Control	25	117.20	6.640	-4.274	N/A	N/A	**.039**
	Dyslexia	19	121.47	6.561		N/A	N/A	(UTT)
**I1**	Control	25	134.64	6.563	-7.044	N/A	N/A	**.002**
	Dyslexia	19	141.68	7.454		N/A	N/A	(UTT)
**I2**	Control	25	110.96	6.181	-3.040	N/A	N/A	.123
	Dyslexia	19	114.00	6.566		N/A	N/A	(UTT)

Table S3. Thickness comparison between groups for the middle retinal layers (INL+OPL+ONL) in the ETDRS grid. Right and left eyes were separately compared. T=Temporal, N=nasal, S=superior, I=Inferior, C0=fovea. Number 1 and number 2 refer to the inner ring and the outer ring, respectively, and correspond to the parafovea (inner ring) and the perifovea (outer ring). n=number, Std. Dev=standard deviation, Dif.=Difference, IQR=interquartile range, UTT= Unpaired t-test MWT=Mann-Whitney test, N/A=not applicable. The thickness results are expressed as microns. Statistically significant results are depicted in bold.

Outer plexiform layer + Outer nuclear layer (OPL+ONL) Comparisons between groups (Right eyes)								
	**Group**	**n**	**Mean**	**Std. dev.**	**Mean Dif.**	**Median**	**IQR**	**Significance (test)**
**C0**	Control	24	110.17	6.391	-6.024	N/A	N/A	**.015**
	Dyslexia	21	116.19	9.506		N/A	N/A	(UTT)
**N1**	Control	24	101.00	6.481	-8.048	N/A	N/A	**.001**
	Dyslexia	21	109.05	8.564		N/A	N/A	(UTT)
**N2**	Control	24	84.88	5.818	-3.887	N/A	N/A	.066
	Dyslexia	21	88.76	7.968		N/A	N/A	(UTT)
**S1**	Control	24	98.67	5.483	-7.619	N/A	N/A	**.003**
	Dyslexia	21	106.29	10.110		N/A	N/A	(UTT)
**S2**	Control	24	86.96	5.196	-2.518	N/A	N/A	.160
	Dyslexia	21	89.48	6.615		N/A	N/A	(UTT)
**T1**	Control	24	98.25	5.995	-5.940	N/A	N/A	**.002**
	Dyslexia	21	104.19	6.298		N/A	N/A	(UTT)
**T2**	Control	24	82.50	5.275	-3.786	N/A	N/A	**.019**
	Dyslexia	21	86.29	5.081		N/A	N/A	(UTT)
**I1**	Control	24	94.83	6.141	-5.024	N/A	N/A	**.012**
	Dyslexia	21	99.86	6.762		N/A	N/A	(UTT)
**I2**	Control	24	77.96	5.369	-3.042	N/A	N/A	.082
	Dyslexia	21	81.00	6.075		N/A	N/A	(UTT)
**Outer plexiform layer + Outer nuclear layer (OPL+ONL) Comparisons between groups (Left eyes)**								
	**Group**	**n**	**Mean**	**Std. dev.**	**Mean Dif.**	**Median**	**IQR**	**Significance (test)**
**C0**	Control	25	109.28	6.471	-6.141	N/A	N/A	**.018**
	Dyslexia	19	115.42	9.985		N/A	N/A	(UTT)
**N1**	Control	25	100.88	6.790	-5.857	N/A	N/A	**.017**
	Dyslexia	19	106.74	8.837		N/A	N/A	(UTT)
**N2**	Control	25	85.16	5.437	-2.366	N/A	N/A	.236
	Dyslexia	19	87.53	7.618		N/A	N/A	(UTT)
**S1**	Control	25	99.68	5.879	-4.952	N/A	N/A	**.017**
	Dyslexia	19	104.63	7.289		N/A	N/A	(UTT)
**S2**	Control	25	87.00	5.083	-3.368	N/A	N/A	.065
	Dyslexia	19	90.37	6.718		N/A	N/A	(UTT)
**T1**	Control	25	98.08	5.575	-5.025	N/A	N/A	**.011**
	Dyslexia	19	103.11	6.887		N/A	N/A	(UTT)
**T2**	Control	25	82.88	5.167	-3.120	N/A	N/A	.058
	Dyslexia	19	86.00	5.364		N/A	N/A	(UTT)
**I1**	Control	25	95.16	6.860	-4.893	N/A	N/A	**.025**
	Dyslexia	19	100.05	7.012		N/A	N/A	(UTT)
**I2**	Control	25	78.04	5.519	-2.855	N/A	N/A	.092
	Dyslexia	19	80.89	5.332		N/A	N/A	(UTT)

Table S4. Thickness comparison between groups for the outer plexiform layer + outer nuclear layer in the ETDRS grid. Right and left eyes were independently compared. T=temporal, N=nasal, S=superior, I=inferior, C0=fovea. Number 1 and number 2 refer to the inner ring and the outer ring, respectively, and correspond to the parafovea (inner ring) and the perifovea (outer ring). n=number, Std. Dev=standard deviation, Dif.=Difference, IQR=interquartile range, UTT= Unpaired t-test MWT=Mann-Whitney test, N/A=not applicable. The thickness results are expressed as microns. Statistically significant results are depicted in bold.

Inner plexiform layer (IPL). Comparisons between groups (Right eyes)								
	**Group**	**n**	**Mean**	**Std. dev.**	**Mean Dif.**	**Median**	**IQR**	**Significance (test)**
**C0**	Control	24	N/A	N/A	N/A	19.00	6	.864 (MWT)
	Dyslexia	21	19.95	3.339		20.00	5	
**N1**	Control	24	41.50	2.467	-1.357	N/A	N/A	.177 (UTT)
	Dyslexia	21	42.86	3.877		N/A	N/A	
**N2**	Control	24	30.46	2.105	-.494	N/A	N/A	.591 (UTT)
	Dyslexia	21	30.95	3.681		N/A	N/A	
**S1**	Control	24	41.13	1.895	N/A	41.00	7	.520 (MWT)
	Dyslexia	21	N/A	N/A		42.00	8	
**S2**	Control	24	28.58	2.394	-.655	N/A	N/A	.419 (UTT)
	Dyslexia	21	29.24	2.982		N/A	N/A	
**T1**	Control	24	N/A	N/A	N/A	41.00	7	.440 (MWT)
	Dyslexia	21	41.19	3.010		42.00	11	
**T2**	Control	24	31.83	2.278	-.738	N/A	N/A	.330 (UTT)
	Dyslexia	21	32.57	2.749		N/A	N/A	
**I1**	Control	24	41.33	1.465	-1.190	N/A	N/A	**.034** (UTT)
	Dyslexia	21	42.52	2.159		N/A	N/A	
**I2**	Control	24	27.67	2.160	-.333	N/A	N/A	.692 (UTT)
	Dyslexia	21	28.00	3.391		N/A	N/A	
**Inner plexiform layer (IPL). Comparisons between groups (Left eyes)**								
	**Group**	**n**	**Mean**	**Std. dev.**	**Mean Dif.**	**Median**	**IQR**	**Significance (test)**
**C0**	Control	25	N/A	N/A	N/A	19.00	5	.895 (MWT)
	Dyslexia	19	20.00	3.091		20.00	4	
**N1**	Control	25	41.88	2.386	-1.962	N/A	N/A	**.013** (UTT)
	Dyslexia	19	43.84	2.588		N/A	N/A	
**N2**	Control	25	N/A	N/A	N/A	31.00	7	.187 (MWT)
	Dyslexia	19	31.42	3.254		32.00	12	
**S1**	Control	25	41.48	1.917	-1.099	N/A	N/A	.120 (UTT)
	Dyslexia	19	42.58	2.673		N/A	N/A	
**S2**	Control	25	28.68	2.340	-1.057	N/A	N/A	.199 (UTT)
	Dyslexia	19	29.74	3.034		N/A	N/A	
**T1**	Control	25	40.44	1.710	-1.981	N/A	N/A	**.009** (UTT)
	Dyslexia	19	42.42	2.694		N/A	N/A	
**T2**	Control	25	31.80	2.062	-.989	N/A	N/A	.180 (UTT)
	Dyslexia	19	32.79	2.760		N/A	N/A	
**I1**	Control	25	41.04	2.189	-1.171	N/A	N/A	.123 (UTT)
	Dyslexia	19	42.21	2.740		N/A	N/A	
**I2**	Control	25	N/A	N/A	N/A	28.00	4	.885 (MWT)
	Dyslexia	19	27.89	2.601		28.00	3	

Table S5. Thickness comparison between groups for the inner plexiform layer in the ETDRS grid. Right and left eyes were independently compared. T=temporal, N=nasal, S=superior, I=inferior, C0=fovea. Number 1 and number 2 refer to the inner ring and the outer ring, respectively, and correspond to the parafovea (inner ring) and the perifovea (outer ring). n=number, Std. Dev=standard deviation, Dif.=Difference, IQR=interquartile range, UTT= Unpaired t-test MWT=Mann-Whitney test, N/A=not applicable. The thickness results are expressed as microns. Statistically significant results are depicted in bold.

Inner nuclear layer (INL). Comparisons between groups (Right eyes)								
	**Group**	**n**	**Mean**	**Std. dev.**	**Mean Dif.**	**Median**	**IQR**	**Significance (test)**
**C0**	Control	24	16.63	3.899	-.327	N/A	N/A	.789 (UTT)
	Dyslexia	21	16.95	4.260		N/A	N/A	
**N1**	Control	24	38.88	2.833	-1.887	N/A	N/A	**.035** (UTT)
	Dyslexia	21	40.76	2.982		N/A	N/A	
**N2**	Control	24	35.67	1.971	N/A	35.50	4	.055 (MWT)
	Dyslexia	21	N/A	N/A		37.00	4	
**S1**	Control	24	39.38	2.901	-1.815	N/A	N/A	**.036** (UTT)
	Dyslexia	21	41.19	2.695		N/A	N/A	
**S2**	Control	24	32.71	1.989	-.911	N/A	N/A	.183 (UTT)
	Dyslexia	21	33.62	2.519		N/A	N/A	
**T1**	Control	24	37.71	2.758	-1.815	N/A	N/A	.052 (UTT)
	Dyslexia	21	39.52	3.341		N/A	N/A	
**T2**	Control	24	34.54	2.146	N/A	35.00	3	.124 (MWT)
	Dyslexia	21	N/A	N/A		35.00	3	
**I1**	Control	24	40.54	3.413	-1.696	N/A	N/A	.094 (UTT)
	Dyslexia	21	42.24	3.208		N/A	N/A	
**I2**	Control	24	33.08	2.358	N/A	33.00	4	.075 (MWT)
	Dyslexia	21	N/A	N/A		34.00	3	
**Inner nuclear layer (INL). Comparisons between groups (Left eyes)**								
	**Group**	**n**	**Mean**	**Std. dev.**	**Mean Dif.**	**Median**	**IQR**	**Significance (test)**
**C0**	Control	25	17.16	4.190	.949	N/A	N/A	.446 (UTT)
	Dyslexia	19	16.21	3.867		N/A	N/A	
**N1**	Control	25	N/A	N/A	N/A	38.00	4	**.017** (MWT)
	Dyslexia	19	40.84	2.651		41.00	6	
**N2**	Control	25	N/A	N/A	N/A	36.00	9	**.024** (MWT)
	Dyslexia	19	37.11	2.558		37.00	4	
**S1**	Control	25	39.44	3.070	-1.771	N/A	N/A	.072 (UTT)
	Dyslexia	19	41.21	3.259		N/A	N/A	
**S2**	Control	25	33.44	2.678	-.507	N/A	N/A	.512 (UTT)
	Dyslexia	19	33.95	2.297		N/A	N/A	
**T1**	Control	25	N/A	N/A	N/A	37.00	11	.132 (MWT)
	Dyslexia	19	38.79	2.992		38.00	4	
**T2**	Control	25	34.32	2.495	-1.154	N/A	N/A	.113 (UTT)
	Dyslexia	19	35.47	2.118		N/A	N/A	
**I1**	Control	25	N/A	N/A	N/A	39.00	4	**.006** (MWT)
	Dyslexia	19	41.63	2.432		42.00	4	
**I2**	Control	25	32.92	2.783	-.185	N/A	N/A	.805 (UTT)
	Dyslexia	19	33.11	1.912		N/A	N/A	

Table S6. Thickness comparison between groups for the inner nuclear layer in the ETDRS grid. Right and left eyes were independently compared. T=temporal, N=nasal, S=superior, I=inferior, C0=fovea. Number 1 and number 2 refer to the inner ring and the outer ring, respectively, and correspond to the parafovea (inner ring) and the perifovea (outer ring). n=number, Std. Dev=standard deviation, Dif.=Difference, IQR=interquartile range, UTT= Unpaired t-test MWT=Mann-Whitney test, N/A=not applicable. The thickness results are expressed as microns. Statistically significant results are depicted in bold.

Inner plexiform layer + Inner nuclear layer (IPL + INL). Comparisons between groups (Right eyes)								
	**Group**	**n**	**Mean**	**Std. dev.**	**Mean Dif.**	**Median**	**IQR**	**Significance (test)**
**C0**	Control	24	36.96	7.445	.054	N/A	N/A	.980 (UTT)
	Dyslexia	21	36.90	6.848		N/A	N/A	
**N1**	Control	24	80.38	4.499	-3.244	N/A	N/A	**.026** (UTT)
	Dyslexia	21	83.62	4.934		N/A	N/A	
**N2**	Control	24	66.13	3.722	-2.351	N/A	N/A	.112 (UTT)
	Dyslexia	21	68.48	5.887		N/A	N/A	
**S1**	Control	24	80.50	4.075	-1.738	N/A	N/A	.228 (UTT)
	Dyslexia	21	82.24	5.431		N/A	N/A	
**S2**	Control	24	61.29	3.793	-1.565	N/A	N/A	.254 (UTT)
	Dyslexia	21	62.86	5.247		N/A	N/A	
**T1**	Control	24	78.38	3.704	-2.339	N/A	N/A	.065 (UTT)
	Dyslexia	21	80.71	4.584		N/A	N/A	
**T2**	Control	24	66.38	3.910	-1.625	N/A	N/A	.216 (UTT)
	Dyslexia	21	68.00	4.775		N/A	N/A	
**I1**	Control	24	81.88	4.100	-2.887	N/A	N/A	**.033** (UTT)
	Dyslexia	21	84.76	4.711		N/A	N/A	
**I2**	Control	24	60.75	4.035	-1.774	N/A	N/A	.218 (UTT)
	Dyslexia	21	62.52	5.446		N/A	N/A	
**Inner plexiform layer + Inner nuclear layer (IPL + INL). Comparisons between groups (Left eyes)**								
	**Group**	**n**	**Mean**	**Std. dev.**	**Mean Dif.**	**Median**	**IQR**	**Significance (test)**
**C0**	Control	25	37.44	7.018	1.229	N/A	N/A	.551 (UTT)
	Dyslexia	19	36.21	6.294		N/A	N/A	
**N1**	Control	25	80.92	4.847	-3.764	N/A	N/A	**.011** (UTT)
	Dyslexia	19	84.68	4.347		N/A	N/A	
**N2**	Control	25	65.88	3.180	-2.646	N/A	N/A	.066 (UTT)
	Dyslexia	19	68.53	5.337		N/A	N/A	
**S1**	Control	25	80.92	3.989	-2.869	N/A	N/A	**.036** (UTT)
	Dyslexia	19	83.79	4.803		N/A	N/A	
**S2**	Control	25	62.12	4.567	-1.564	N/A	N/A	.279 (UTT)
	Dyslexia	19	63.68	4.831		N/A	N/A	
**T1**	Control	25	78.20	4.193	-3.011	N/A	N/A	**.018** (UTT)
	Dyslexia	19	81.21	3.750		N/A	N/A	
**T2**	Control	25	66.12	4.186	-2.143	N/A	N/A	.111 (UTT)
	Dyslexia	19	68.26	4.507		N/A	N/A	
**I1**	Control	25	80.52	4.436	-3.322	N/A	N/A	**.020** (UTT)
	Dyslexia	19	83.84	4.598		N/A	N/A	
**I2**	Control	25	60.52	4.032	-.480	N/A	N/A	.706 (UTT)
	Dyslexia	19	61.00	4.308		N/A	N/A	

Table S7. Thickness comparison between groups for the Inner plexiform layer + Inner nuclear layer in the ETDRS grid. Right and left eyes were independently compared. T=temporal, N=nasal, S=superior, I=inferior, C0=fovea. Number 1 and number 2 refer to the inner ring and the outer ring, respectively, and correspond to the parafovea (inner ring) and the perifovea (outer ring). n=number, Std. Dev=standard deviation, Dif.=Difference, IQR=interquartile range, UTT= Unpaired t-test MWT=Mann-Whitney test, N/A=not applicable. The thickness results are expressed as microns. Statistically significant results are depicted in bold.

Retinal nerve fiber layer (RNFL). Comparisons between groups (Right eyes)								
	**Group**	**n**	**Mean**	**Std. dev.**	**Mean Dif.**	**Median**	**IQR**	**Significance (test)**
**C0**	Control	24	N/A	N/A	N/A	11.0	4	.645 (MWT)
	Dyslexia	21	10.86	2.151		11.0	3	
**N1**	Control	24	N/A	N/A	N/A	19	3	.316 (MWT)
	Dyslexia	21	19.00	2.049		19	3	
**N2**	Control	24	46.71	6.963	1.851	N/A	N/A	.339 (UTT)
	Dyslexia	21	44.86	5.712		N/A	N/A	
**S1**	Control	24	22.54	2.206	.113	N/A	N/A	.864 (UTT)
	Dyslexia	21	22.43	2.181		N/A	N/A	
**S2**	Control	24	34.33	5.983	-.476	N/A	N/A	.751 (UTT)
	Dyslexia	21	34.81	3.544		N/A	N/A	
**T1**	Control	24	16.08	1.018	.607	N/A	N/A	.085 (UTT)
	Dyslexia	21	15.48	1.289		N/A	N/A	
**T2**	Control	24	17.46	1.285	.363	N/A	N/A	.338 (UTT)
	Dyslexia	21	17.10	1.221		N/A	N/A	
**I1**	Control	24	N/A	N/A	N/A	24	5	.201 (MWT)
	Dyslexia	21	23.19	2.482		23	3	
**I2**	Control	24	N/A	N/A	N/A	38.5	11	.236 (MWT)
	Dyslexia	21	37.29	4.849		37.0	7	
**Retinal nerve fiber layer (RNFL). Comparisons between groups (Left eyes)**								
	**Group**	**n**	**Mean**	**Std. dev.**	**Mean Dif.**	**Median**	**IQR**	**Significance (test)**
**C0**	Control	25	N/A	N/A	N/A	11.0	3	.799 (MWT)
	Dyslexia	19	11.05	2.013		11.0	2	
**N1**	Control	25	19.48	1.447	.674	N/A	N/A	.703 (UTT)
	Dyslexia	19	19.42	1.644		N/A	N/A	
**N2**	Control	25	46.20	5.553	-.215	N/A	N/A	.769 (UTT)
	Dyslexia	19	45.53	6.040		N/A	N/A	
**S1**	Control	25	21.68	2.545	.533	N/A	N/A	.708 (UTT)
	Dyslexia	19	21.89	2.158		N/A	N/A	
**S2**	Control	25	34.48	5.001	.008	N/A	N/A	.975 (UTT)
	Dyslexia	19	33.95	4.102		N/A	N/A	
**T1**	Control	25	N/A	N/A	N/A	16.0	1	.759 (MWT)
	Dyslexia	19	15.63	1.012		16.0	1	
**T2**	Control	25	N/A	N/A	N/A	17.0	1	.054 (MWT)
	Dyslexia	19	N/A	N/A		17.0	1	
**I1**	Control	25	N/A	N/A	N/A	25.0	2	.341 (MWT)
	Dyslexia	19	23.32	2.029		24.0	3	
**I2**	Control	25	37.60	6.124	.059	N/A	N/A	.900 (UTT)
	Dyslexia	19	36.42	4.376		N/A	N/A	

Table S8. Thickness comparison between groups for the retinal nerve fiber layer (RNFL) in the ETDRS grid. Right and left eyes were independently compared. T=temporal, N=nasal, S=superior, I=inferior, C0=fovea. Number 1 and number 2 refer to the inner ring and the outer ring, respectively, and correspond to the parafovea (inner ring) and the perifovea (outer ring). n=number, Std. Dev=standard deviation, Dif.=Difference, IQR=interquartile range, UTT= Unpaired t-test MWT=Mann-Whitney test, N/A=not applicable. The thickness results are expressed as microns.

Ganglion cell layer. Comparisons between groups (Right eyes)								
	**Group**	**n**	**Mean**	**Std. dev.**	**Mean Dif.**	**Median**	**IQR**	**Significance (test)**
**C0**	Control	24	N/A	N/A	N/A	13.5	7	.846 (MWT)
	Dyslexia	21	14.33	4.066		15	6	
**N1**	Control	24	51.92	3.550	-0.32	N/A	N/A	.804 (UTT)
	Dyslexia	21	52.24	5.029		N/A	N/A	
**N2**	Control	24	40.13	2.787	0.17	N/A	N/A	.895 (UTT)
	Dyslexia	21	39.95	5.315		N/A	N/A	
**S1**	Control	24	53.71	3.071	N/A	53	4	.846 (MWT)
	Dyslexia	21	N/A	N/A		55	5	
**S2**	Control	24	35.83	3.422	-0.31	N/A	N/A	.782 (UTT)
	Dyslexia	21	36.14	4.028		N/A	N/A	
**T1**	Control	24	49.75	3.096	0.94	N/A	N/A	.394 (UTT)
	Dyslexia	21	48.81	4.214		N/A	N/A	
**T2**	Control	24	37.21	4.170	0.54	N/A	N/A	.697 (UTT)
	Dyslexia	21	36.67	5.083		N/A	N/A	
**I1**	Control	24	N/A	N/A	N/A	53	3	.463 (MWT)
	Dyslexia	21	53.86	3.785		54	6	
**I2**	Control	24	34.75	2.953	-0.16	N/A	N/A	.891 (UTT)
	Dyslexia	21	34.90	4.482		N/A	N/A	
**Ganglion cell layer. Comparisons between groups (Left eyes)**								
	**Group**	**n**	**Mean**	**Std. dev.**	**Mean Dif.**	**Median**	**IQR**	**Significance (test)**
**C0**	Control	25	N/A	N/A	N/A	13	7	.398 (MWT)
	Dyslexia	19	14.16	4.337		13	7	
**N1**	Control	25	52.04	3.310	-0.59	N/A	N/A	.603 (UTT)
	Dyslexia	19	52.63	4.193		N/A	N/A	
**N2**	Control	25	39.96	2.638	-0.83	N/A	N/A	.477 (UTT)
	Dyslexia	19	40.79	4.454		N/A	N/A	
**S1**	Control	25	53.36	3.147	N/A	53	5	.520 (MWT)
	Dyslexia	19	N/A	N/A		54	6	
**S2**	Control	25	36.28	3.129	-0.30	N/A	N/A	.773 (UTT)
	Dyslexia	19	36.58	3.687		N/A	N/A	
**T1**	Control	25	48.88	3.308	-0.38	N/A	N/A	.721 (UTT)
	Dyslexia	19	49.26	3.754		N/A	N/A	
**T2**	Control	25	36.52	3.959	0.20	N/A	N/A	.872 (UTT)
	Dyslexia	19	36.32	4.334		N/A	N/A	
**I1**	Control	25	N/A	N/A	N/A	53	4	.691 (MWT)
	Dyslexia	19	53.47	4.060		53	6	
**I2**	Control	25	34.80	3.240	0.27	N/A	N/A	.794 (UTT)
	Dyslexia	19	34.53	3.642		N/A	N/A	

Table S9. Thickness comparison between groups for the ganglion cell layer (GCL) in the ETDRS grid. Right and left eyes were independently compared. T=temporal, N=nasal, S=superior, I=inferior, C0=fovea. Number 1 and number 2 refer to the inner ring and the outer ring, respectively, and correspond to the parafovea (inner ring) and the perifovea (outer ring). n=number, Std. Dev=standard deviation, Dif.=Difference, IQR=interquartile range, UTT= Unpaired t-test MWT=Mann-Whitney test, N/A=not applicable. The thickness results are expressed as microns.

Ganglion Cell Complex. Comparisons between groups (Right eye)								
	**Group**	**n**	**Mean**	**Std. dev.**	**Mean Dif.**	**Median**	**IQR**	**Significance (test)**
**C0**	Control	24	N/A	N/A	N/A	43.50	17	.802
	Dyslexia	21	45.14	9.172		46	13	(MWT)
**N1**	Control	24	113.21	7.089	-.887	N/A	N/A	.725
	Dyslexia	21	114.10	9.648		N/A	N/A	(UTT)
**N2**	Control	24	117.29	8.888	1.530	N/A	N/A	.632
	Dyslexia	21	115.76	12.316		N/A	N/A	(UTT)
**S1**	Control	24	117.38	6.149	N/A	115	8	.516
	Dyslexia	21	N/A	N/A		119	10	(MWT)
**S2**	Control	24	98.75	10.427	-1.440	N/A	N/A	.630
	Dyslexia	21	100.19	9.315		N/A	N/A	(UTT)
**T1**	Control	24	106.50	4.364	N/A	106	6	.710
	Dyslexia	21	N/A	N/A		108	14	(MWT)
**T2**	Control	24	86.50	6.200	.167	N/A	N/A	.935
	Dyslexia	21	86.33	7.519		N/A	N/A	(UTT)
**I1**	Control	24	N/A	N/A	N/A	117	8	.732
	Dyslexia	21	119.57	7.018		120	7	(MWT)
**I2**	Control	24	103.17	10.458	2.976	N/A	N/A	.353
	Dyslexia	21	100.19	10.792		N/A	N/A	(UTT)
**Ganglion Cell Complex. Comparisons between groups (Left eye)**								
	**Group**	**n**	**Mean**	**Std. dev.**	**Mean Dif.**	**Median**	**IQR**	**Significance (test)**
**C0**	Control	25	N/A	N/A	N/A	43	15	.661
	Dyslexia	19	45.14	9.172		44	14	(MWT)
**N1**	Control	25	113.21	7.089	-.887	N/A	N/A	.225
	Dyslexia	19	114.10	9.648		N/A	N/A	(UTT)
**N2**	Control	25	117.29	8.888		116	14	.991
	Dyslexia	19	N/A	N/A	N/A	115	21	(MWT)
**S1**	Control	25	117.38	6.149	1.327	N/A	N/A	.327
	Dyslexia	19	116.05	11.066		N/A	N/A	(UTT)
**S2**	Control	25	98.75	10.427	-1.440	N/A	N/A	.770
	Dyslexia	19	100.19	9.315		N/A	N/A	(UTT)
**T1**	Control	25	106.50	4.364	1.024	N/A	N/A	.130
	Dyslexia	19	105.48	7.339		N/A	N/A	(UTT)
**T2**	Control	25	N/A	N/A	N/A	84	9	.484
	Dyslexia	19	86.33	7.519		86	13	(MWT)
**I1**	Control	25	120.42	8.293	.845	N/A	N/A	.721
	Dyslexia	19	119.57	7.018		N/A	N/A	(UTT)
**I2**	Control	25	103.17	10.458	2.976	N/A	N/A	.652
	Dyslexia	19	100.19	10.792		N/A	N/A	(UTT)

Table S10. Thickness comparison between groups for the ganglion cell complex (GCC) in the ETDRS grid. Right and left eyes were independently compared. T=temporal, N=nasal, S=superior, I=inferior, C0=fovea. Number 1 and number 2 refer to the inner ring and the outer ring, respectively, and correspond to the parafovea (inner ring) and the perifovea (outer ring). n=number, Std. Dev=standard deviation, Dif.=Difference, IQR=interquartile range, UTT= Unpaired t-test MWT=Mann-Whitney test, N/A=not applicable. The thickness results are expressed as microns.

Outer retinal. Comparisons between groups (Right eyes)								
	Group	n	Mean	Std. dev.	Mean Dif.	Median	IQR	Significance (test)
C0	Control	24	87.33	3.158	1.476	N/A	N/A	.116 (UTT)
	Dyslexia	21	85.86	2.988		N/A	N/A	
N1	Control	24	80.83	2.014	.119	N/A	N/A	.843 (UTT)
	Dyslexia	21	80.71	1.978		N/A	N/A	
N2	Control	24	78.17	1.786	-.167	N/A	N/A	.775 (UTT)
	Dyslexia	21	78.33	2.106		N/A	N/A	
S1	Control	24	79.38	2.584	.423	N/A	N/A	.536 (UTT)
	Dyslexia	21	78.95	1.830		N/A	N/A	
S2	Control	24	77.25	1.984	-.702	N/A	N/A	.246 (UTT)
	Dyslexia	21	77.95	2.012		N/A	N/A	
T1	Control	24	79.17	2.531	.452	N/A	N/A	.488 (UTT)
	Dyslexia	21	78.71	1.648		N/A	N/A	
T2	Control	24	76.38	2.102	-.149	N/A	N/A	.787 (UTT)
	Dyslexia	21	76.52	1.470		N/A	N/A	
I1	Control	24	78.54	2.064	.208	N/A	N/A	.706 (UTT)
	Dyslexia	21	78.33	1.528		N/A	N/A	
I2	Control	24	75.83	1.786	N/A	76	3	.148 (MWT)
	Dyslexia	21	N/A	N/A		77	2	
Outer retina. Comparisons between groups (Left eyes)								
	Group	n	Mean	Std. dev.	Mean Dif.	Median	IQR	Significance (test)
C0	Control	25	88.48	4.464	2.585	N/A	N/A	.051 (UTT)
	Dyslexia	19	85.89	3.740		N/A	N/A	
N1	Control	25	81.88	2.934	1.459	N/A	N/A	.069 (UTT)
	Dyslexia	19	80.42	1.981		N/A	N/A	
N2	Control	25	78.48	1.735	N/A	79	3	.238 (MWT)
	Dyslexia	19	N/A	N/A		78	4	
S1	Control	25	79.28	2.654	.701	N/A	N/A	.358 (UTT)
	Dyslexia	19	78.58	2.219		N/A	N/A	
S2	Control	25	N/A	N/A	N/A	78	3	.680 (MWT)
	Dyslexia	19	77.63	2.033		78	3	
T1	Control	25	79.52	2.485	N/A	80	4	.256 (MWT)
	Dyslexia	19	N/A	N/A		79	3	
T2	Control	25	76.44	2.219	.124	N/A	N/A	.853 (UTT)
	Dyslexia	19	76.32	2.136		N/A	N/A	
I1	Control	25	78.80	2.449	.800	N/A	N/A	.242 (UTT)
	Dyslexia	19	78.00	1.856		N/A	N/A	
I2	Control	25	76.08	1.605	N/A	76	2	.838 (MWT)
	Dyslexia	19	N/A	N/A		76	2	

Table S11. Thickness comparison between groups for outer retina in the ETDRS grid. Right and left eyes were independently compared. T=temporal, N=nasal, S=superior, I=inferior, C0=fovea. Number 1 and number 2 refer to the inner ring and the outer ring, respectively, and correspond to the parafovea (inner ring) and the perifovea (outer ring). n=number, Std. Dev=standard deviation, Dif.=Difference, IQR=interquartile range, UTT= Unpaired t-test MWT=Mann-Whitney test, N/A=not applicable. The thickness results are expressed as microns.
